# Revealing the effect of electrocatalytic performance boost during hydrogen evolution reaction on free-standing SWCNT film electrode

**DOI:** 10.1038/s41598-021-99458-8

**Published:** 2021-10-07

**Authors:** Karolina Kordek-Khalil, Dawid Janas, Piotr Rutkowski

**Affiliations:** 1 Department of Process Engineering and Technology of Polymer and Carbon Materials, Wrocław University of Science and Technology, Wybrzeże Wyspiańskiego 27, 50-370 Wrocław, Poland; 2 Department of Organic Chemistry, Bioorganic Chemistry and Biotechnology, Silesian University of Technology, B. Krzywoustego 4, 44-100 Gliwice, Poland

**Keywords:** Carbon nanotubes and fullerenes, Electronic properties and materials

## Abstract

Large-scale sustainable hydrogen production by water electrolysis requires a highly active yet low-cost hydrogen evolution reaction (HER) electrocatalyst. Conductive carbon nanomaterials with high surface areas are promising candidates for this purpose. In this contribution, single-walled carbon nanotubes (SWCNTs) are assembled into free-standing films and directly used as HER electrodes. During the initial 20 h of electrocatalytic performance in galvanostatic conditions, the films undergo activation, which results in a gradual overpotential decrease to the value of 225 mV. Transient physicochemical properties of the films at various activation stages are characterized to reveal the material features responsible for the activity boost. Results indicate that partial oxidation of iron nanoparticles encapsulated in SWCNTs is the major contributor to the activity enhancement. Furthermore, besides high activity, the material, composed of only earth-abundant elements, possesses exceptional performance stability, with no activity loss for 200 h of galvanostatic performance at − 10 mA cm^−2^. In conclusion, the work presents the strategy of engineering a highly active HER electrode composed of widely available elements and provides new insights into the origins of electrocatalytic performance of SWCNT-based materials in alkaline HER.

## Introduction

In the emerging era of large-scale production of fuel cell vehicles and successively growing market of fuel cell combined heat and power (CHP) systems, developing both low-cost and zero-emission methods of hydrogen production is a critical issue^1,2^. Currently, hydrogen is produced on a large scale by steam reforming of natural gas, partial oxidation of crude oil and coal gasification^3^. Nevertheless, if the zero-emission scenario is to be implemented, this gas generation must become independent from fossil fuels. Water is an attractive and renewable raw material for hydrogen production. Among methods of its splitting, alkaline water electrolysis (AWE) is the most mature technology, with a strong advantage of the formation of very pure hydrogen, which can be directly applied in hydrogen fuel cells^4^.

Nowadays, the biggest limitation of the AWE is the high cost of electricity required to run the process. Especially, high contribution to the total operating voltage is made by high activation energies of two water splitting half-reactions of hydrogen evolution reaction (HER) and oxygen evolution reaction (OER)^5,6^. Minimisation of the reaction overpotentials can be achieved by using proper electrocatalysts. Platinum-based materials so far exhibit the highest electrocatalytic activities in HER at present^7,8^. However, the scarcity and cost of platinum prevent their large-scale application and evokes intense research efforts targeted to study HER electrocatalytic properties in noble-metal free materials^9^.

Recently, various carbon (nano)materials and their composites with non-noble metals (especially nickel and cobalt) emerged as a promising class of highly active HER electrocatalysts. For example, high performance in alkaline HER was achieved using Ni_3_Fe nanoparticles combined with N-doped carbon nanotube–grafted carbon nanofibers (CNFs)^10^, Ni–Mo–N nanoparticles decorated on reduced graphene oxide (rGO)^11^, and Fe_3_C-Co nanoparticles encapsulated in N-doped carbon^12^. These materials offer low HER overpotentials, which are already competitive. However, it is uncertain whether the electrocatalytic performance of these materials is stable during prolonged operation. The stability tests at current densities of ca. 10 mA cm^−2^ are rarely reported for times longer than 36 h.

Unfortunately, all nanomaterials mentioned above, which are promising for HER, typically come in powder form. Therefore, for testing and operation in an electrochemical setup, it is required to paste them to the electrode surface by polymer binders, which may add additional resistance factors to the system and block electrocatalytically active sites^13^. Moreover, electrocatalyst coatings formed this way often form aggregates with irregular voids, which is not beneficial for gas transport and may impede reaction kinetics or cause materials delamination from the electrode surface upon a vigorous gas evolution^14,15^. This motivates the arrangement of such advanced conductive carbon nanomaterials into free-standing form, which assures both good mass and electron transfer. There is a wide range of techniques for producing such ensembles from carbon nanomaterials e.g. graphene or CNTs^16^. However, most of these methods either give insufficient material for wider implementation or the electrical conductivity of the manufactured nanocarbon networks is not competitive unless the material is doped. Recently, it was shown that single-walled CNTs (SWCNTs) can be arranged in a straightforward and scalable fashion to fabricate thin free-standing films of appreciable electrochemical characteristics^17^.

Admittedly, in the field of electrocatalysis, it is common to analyze materials only before activity tests, ignoring the fact that strongly reactive conditions during electrocatalytic reactions can also cause changes in electrocatalytic material itself^18,19^. In this work, we decided to thoroughly analyze this phenomenon using free-standing SWCNT films of high electrical conductivity as electrodes for HER. First, we investigated the material morphology and composition after HER. Then, we compared it with the properties of raw material to (1) show the particular chemical species responsible for the electrocatalytic activity and (2) identify the changes in the material which provided it with upgraded electrocatalytic performance over time. Besides the gained insight into the mechanics of the process, we demonstrate that the selected material reveals excellent electrochemical activity toward HER with a stable performance at the current density of 10 mA cm^−2^ for over 8 days with the overpotential of 225 mV.

## Experimental

### SWCNT purification

Large-diameter SWCNTs (Tuball™, OCSiAl) were subjected to a purification routine reported below to reduce residual catalyst content^20^. In brief, 250 mg of metallic sodium and 1 390 mg of naphthalene were added to 50 mL of anhydrous dimethylacetamide. Then, the solution was diluted 5×, and raw SWCNTs (250 mg) were introduced. Such a mixture was kept overnight to facilitate the purification. Afterward, it was ultracentrifuged for 1 h (15,314×*g*, Eppendorf Centrifuge 5804 R) to form a pellet and a supernatant. Next, the separated solid part was washed multiple times with acetone and distilled water using vacuum filtration (PTFE filters, pore size: 0.2 μm, Whatman). After the purification strategy conducted in moisture- and air-free glovebox, purified SWCNTs were stored in a desiccator. According to TGA, the processing reduced residual catalyst (Fe) amount from 21.3 to 3.53% (Fig. S1). The remaining small content of Fe is visualized in Fig. S2.

### Material preparation

In order to evaluate the electrochemical properties of purified SWCNTs, free-standing films were manufactured from the purified SWCNT powder by a method described by us before^21^. Briefly, SWCNT dispersion was made in an ice-cold mixture of toluene and acetone (1:1, w/w) using sonication (Hielscher UP200St, Germany) at 100% amplitude. Ethyl cellulose (EC) was employed as a binding agent in a 1:50 ratio with respect to the SWCNTs. The obtained dispersion was deposited onto a Nomex sheet, to which nanocarbon has low adhesion. This reasoning facilitated the delamination of the films from the substrate. Finally, EC was removed by annealing in air, which rapidly eliminated the binder while keeping the properties of the SWCNTs intact. The appearance of the SWCNT films is shown in Fig. S3.

### Material characterization

The amount of residual catalyst in SWCNTs was studied by TGA. Thermograms were recorded in the air (30 mL/min) from 25 to 1000 °C at a 10 °C/min heating rate. 2 mg of material were analyzed per specimen.

The degree of structural perfection of SWCNT films was gauged using Raman spectroscopy (Renishaw inVia system, λ = 514 nm laser, 10% of power). Multiple spectra were acquired within the range from 70 to 3300 cm^−1^ to ensure the acquisition of an appreciable signal-to-noise ratio.

The chemical composition of the material was characterized through X-ray photoelectron spectroscopy (XPS), applying Specs PHOIBOS 150 hemispherical energy analyzer. The binding energy (BE) scale was adjusted by using the peaks of Au 4f_7/2_ (84.0 eV) as a reference. The obtained data was fitted using algorithms embedded in CASA XPS software (version: 2.3.15). The background modeled by the Shirley function was subtracted.

The morphology of SWCNT electrodes was characterized with a Field Emission Scanning Electron Microscope (FE-SEM, Magellan 400L) and a Transmission Electron Microscope (TEM, FEI Tecnai G2 F20). Additionally, Energy-dispersive X-ray spectroscopy (EDX) module coupled with the TEM apparatus was employed to probe the chemical composition of the samples within the 0–10 keV excitation range.

Iron concentration in the electrolyte was evaluated with an Inductively Coupled Plasma Optical Emission Spectrometer (ICP-OES) Agilent 5110 at 238 nm.

### Electrochemical properties

The electrocatalytic activity tests in HER were performed in a standard 3-electrode system using ATLAS 0531 electrochemical unit. In each experiment, the 2 × 1 cm^2^ SWCNT film was directly applied as a working electrode. The Hg/HgO (1.0 M KOH) electrode and graphite rod were used as a reference and a counter electrode, respectively. The long-term performance of the SWCNT film was evaluated by means of chronopotentiometry at current densities of 10 and 100 mA cm^−2^ in an H_2_-saturated 1.0 M KOH solution. The electrocatalytic activity in HER was also assessed by Linear Sweep Voltammetry (LSV). The tested samples included SWCNT film directly after removing the binder and SWCNT film samples after 15 min, 2 h, and 20 h of HER chronopotentiometry tests at 10 mA cm^−2^ (denoted SWCNT-film HER 15’, SWCNT-film HER 2 h and SWCNT-film HER 20 h, respectively). Additionally, samples prepared by removal of binder and following: (1) immersion in KOH for 15 min, 2 h, and 20 h (SWCNT-film KOH 20 h) or (2) storage in the air for 20 h (SWCNT-film air 20 h) were also examined as HER electrocatalysts by LSV. All LSV plots were recorded at a scan rate of 5 mV s^−1^, and potentials were converted with reference to the reversible hydrogen electrode (RHE) according to the following equation: *E*_*RHE*_ = *E*_*Hg/HgO*_ + 0.098 + 0.059*pH* and corrected for iR compensation.

Pt/C (10 wt%, Alfa Aesar) was used as a benchmark electrocatalyst for HER. Due to the powder form of this catalyst, 4 mg of the material was dispersed in a solution composed of 700 µL of water, 250 µL of ethanol, and 50 µL of Nafion. After sonication for 30 min, the working electrode was prepared by drop-casting the dispersions on a glassy carbon electrode surface (GCE) with a diameter of 5 µm. The electrode prepared this way was tested using a typical protocol for the characterization of carbon cloth-based samples.

In the same 3-electrode experimental setup, the electrochemical capacitance of the electrodes was estimated from CV scans in a potential range between − 0.1 and 0.1 V versus Hg/HgO at different scan rates of 20, 40, 60, 80, and 100 mV s^−1^. The capacitive currents measured at 0 V versus Hg/HgO were plotted as a function of a scan rate. Specific capacitances, directly proportional to electrochemically active surface areas, were obtained by linear fitting the plots. Electrochemical impedance spectra (EIS) were acquired in a frequency range from 200 kHz to 10 mHz using an amplitude of 5 mV at HER overpotential of 275 mV. The equivalent circuits were fitted to the Nyquist plots using EC-lab software (version: 11.20). The Faradaic yield (*FY*) of HER was determined by comparison of the gas volume generated during galvanostatic electrolysis at the current density of 5 mA cm^−2^ (*V*_*experminental*_) with the theoretical value (*V*_*theoretical*_), according to the following equation: $FY = \frac{{V}_{experimental}}{{V}_{theoretical}}=\frac{{V}_{experimental}}{\frac{Q}{zF}{V}_{m}}$, where *Q* is the electric charge passed through the substrate, *F* is the Faraday constant, *z* is the number of electrons transferred per ion (equal to 2 for H_2_), and *V*_*m*_ is the molar volume of hydrogen gas (24.5 dm^3^ mol^−1^)^22^.

## Results and discussion

**Figure 1:**
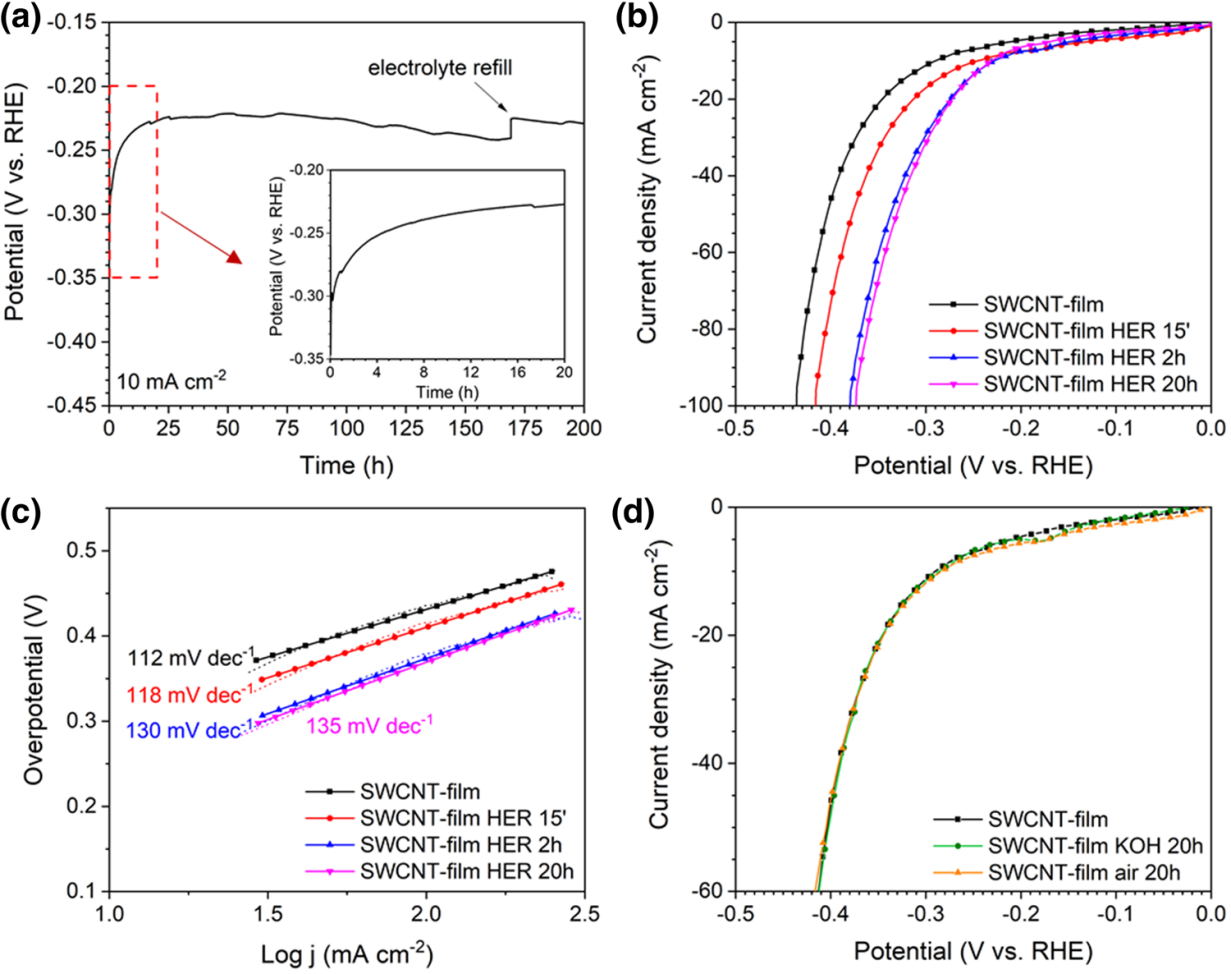
(**a**) Chronopotentiometry curve for freshly annealed SWCNT film at the current density of 10 mA cm^−2^, (**b**) LSV curves for freshly annealed SWCNT film and after 15 min, 2 h, and 20 h of HER at the current density of 10 mA cm^−2^ and (**c**) corresponding Tafel slopes, (**d**) comparison of LSV plots for freshly annealed SWCNT film and after its exposure to air or immersion in 1.0 M KOH for 20 h before the measurement. All experiments were performed in 1.0 M KOH.

Freshly annealed SWCNT film was directly applied as an electrocatalytic electrode for HER in 1.0 M KOH. The electrode performance in galvanostatic electrocatalysis at 10 mA cm^−2^ was tested for a long time of 200 h. The acquired chronopotentiometry plot is presented in Fig. 1a. As can be seen in the inset, the potential required to provide the current density of 10 mA cm^−2^ during HER significantly decreased during the first 20 h of electrocatalyst performance, indicating that the reaction overpotential was substantially reduced from ca. 300 to 225 mV. After this activation period, the reaction overpotential was approximately stable for the following 100 h of electrolysis. Next, a slight progressive overpotential rise was noted, probably caused by electrolyte evaporation, which reduced the surface of the electrocatalyst immersed in the electrolyte. This hypothesis was validated by the return of overpotential to the previous value of approximately 225 mV upon electrolyte refill after ca. 175 h of electrolysis. Besides the discussed phenomenon, the chronopotentiometry test demonstrated excellent material stability for a period of time much longer than usually reported for HER electrocatalysts^13,23,24^. Moreover, the determined overpotential value of 225 mV can be considered attractive for a noble metal-free HER electrocatalyst utilized in an alkaline medium.

We decided to elucidate the observed activity increase in the first 20 h of electrolysis using the LSV technique. Polarization curves for HER in 1.0 M KOH were acquired for the fresh SWCNT films as well as for the materials after different periods of galvanostatic treatment at the current density of 10 mA cm^−2^_._ The LSV plots, presented in Fig. 1b, confirmed that the reaction overpotential gradually decreased during the first 20 h of electrolysis from ca. 290 to 225 mV at 10 mA cm^−2^. The corresponding Tafel slopes, presented in Fig. 1c, were in the range between 112 mV dec^−1^ (fresh SWCNT film) and 135 mV dec^−1^ (SWCNT film after 20 h of HER). The determined values close to 120 mV dec^−1^ strongly suggested that the first stage of HER (the Volmer reaction, according to the equation *H*_2_*O* + *e*^−^ = *H*_*ads*_ + *OH*^−^) was the rate-determining step of the process in the case of all tested electrodes. The trend of the Tafel slope slightly increasing with the electrocatalysis time can be noted. The increased electrocatalyst surface coverage by adsorbed hydrogen during prolonged electrolysis can cause this effect^25^. Moreover, the operational parameters of the SWCNTs films were found to be encouraging. The Faradaic efficiency of the HER process using the SWCNT film HER 20 h sample reached 96% (Fig. S4). Furthermore, this electrode's electrocatalytic activity was satisfactory compared with the performance of benchmark Pt/C electrocatalyst (Fig. S5). While the HER overpotential on SWCNT-film HER 20 h was 125 mV higher than recorded using Pt/C at the current density of 100 mA cm^−2^, such electrodes are flexible, inexpensive, easily recyclable, and can be produced from sustainable substrates^26^.

Additional experiments were carried out to assess if the anomalous material activation resulted from the prolonged exposure of SWCNT films to the experimental conditions. To exclude the possible impact of air or KOH on the electrochemical performance of SWCNTs, freshly annealed films were either subjected to contact with air for 20 h or immersed in KOH for 15 min, 2 h, and 20 h (without providing the reductive potential). The comparison of the LSV plots of the aforementioned samples is presented in Fig. 1d and S6. As can be noted, the course of the presented LSV curves was almost identical to the curve recorded for the fresh samples. Furthermore, to exclude the possibility that the catalytic activity comes from Fe impurities present in the electrolyte, we characterized it by ICP-OES. The fresh 1.0 M KOH had the Fe concentration of 27.3 ppb (2.7 × 10^–6^%), which is infinitesimal compared to the content of Fe in SWCNTs. Thus, Fe from this source may be disregarded. These observation suggested that the activation was initiated by using SWCNT films as a cathode in electrolysis rather than external factors. A spectrum of material characterization techniques was applied in order to explain the material activation phenomenon.

One of the most common ways to gauge the crystallinity of nanocarbon is Raman spectroscopy. The ratio of intensities of the defect-induced band D (at ~ 1330 cm^−1^) to the band of graphitic vibrations G (at ~ 1580 cm^−1^) quantifies the amount of defects and functional groups^27^. The neat SWCNT film was of high quality, as shown by a relatively low I_D_/I_G_ ratio of only 0.05 (Fig. S7). What is more, the presence of the radial-breathing mode (RBM) band proved that the CNTs were single-walled. Lastly, the shape of the G feature (characteristic shoulder at lower wavenumbers) showed that the material was predominantly metallic^28^, which should ensure high electrical conductivity. What is essential, the I_D_/I_G_ ratios remained unchanged by HER (regardless of the reaction time) or air/KOH pre-treatments suggesting that the SWCNTs were not deteriorated by these processes (at all or to a detectable extent).

**Figure 2:**
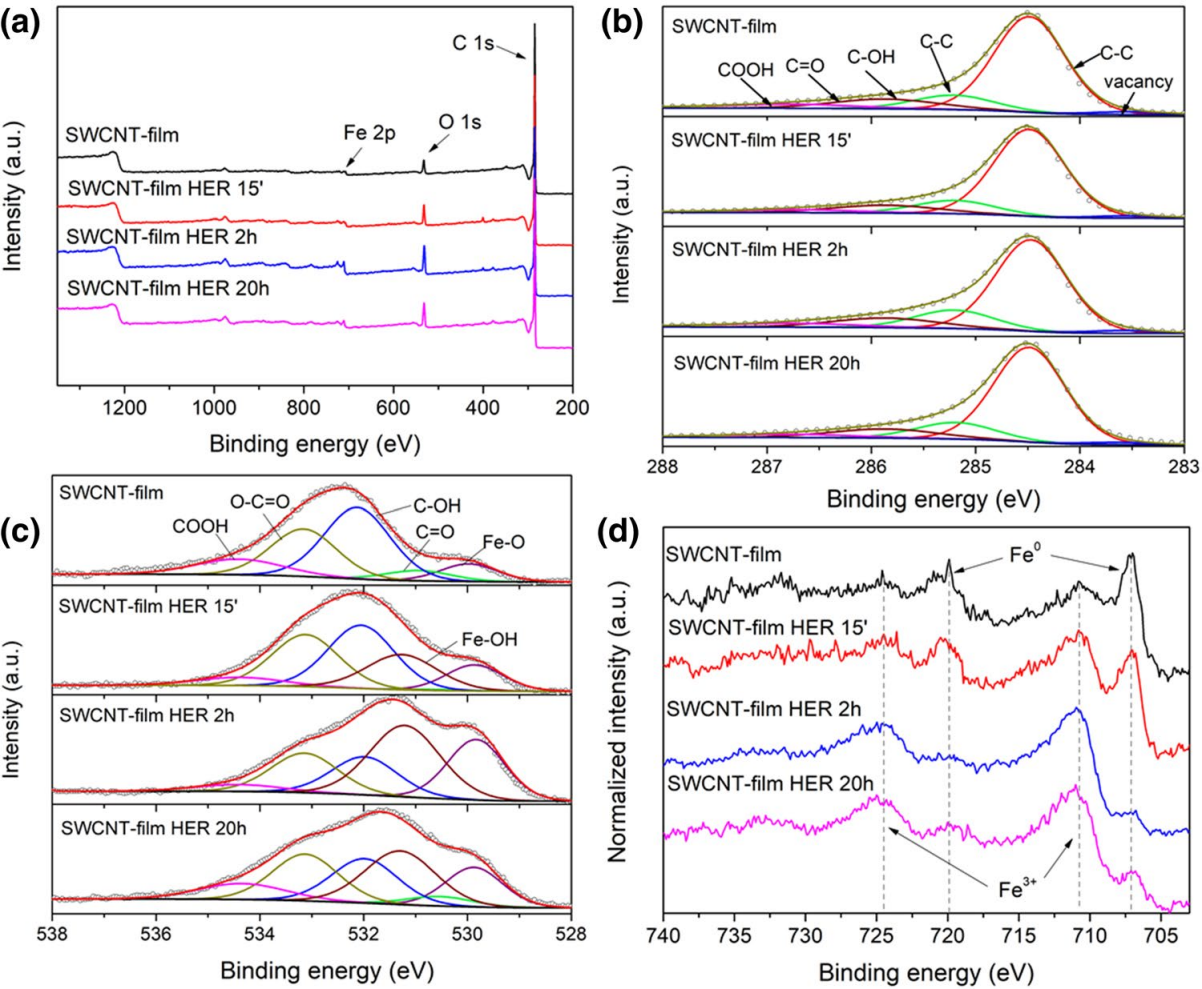
(**a**) XPS survey spectra, and high-resolution XPS spectra in (**b**) C 1s, (**c**) O 1s, and (**d**) Fe 2p regions for freshly annealed SWCNT film and after 15 min, 2 h, and 20 h of HER at the current density of 10 mA cm^−2^.

Since XPS is a more sensitive technique to determine the surface chemical state, it was used to analyze the SWCNT films used for HER. Collected survey spectra, presented in Fig. 2a, revealed that the samples were primarily composed of carbon and oxygen, with small amounts of iron (residual catalyst from the synthesis). Subsequently, high-resolution spectra were acquired, and individual elemental peaks were deconvoluted to study the effect of material activation in more detail. The signal in the C 1s region (Fig. 2b) was resolved into 6 peaks, which were attributed to carbon atoms in the following configurations: delocalized C–C (284.5 eV), localized C–C (285.2 eV), C–OH (285.9 eV), C=O (286.7 eV) and COOH (288.3 eV)^29^. Moreover, the signal located at 285.2 eV was assigned to carbon atoms neighboring the lattice vacancies^30^. The C 1 s spectra of all SWCNT films, regardless of the HER time, are similar, and their deconvolution did not reveal any radical changes in the chemical states of carbon atoms. This result stayed in accordance with earlier findings obtained from Raman spectroscopy (Fig. S1), thereby showing that (1) SWCNTs are remarkably durable when used as an electrode for HER, and (2) the enhancement of electrocatalytic performance is not a result of chemical modification of SWCNTs.

Furthermore, other elements were analyzed to look for an explanation. The deconvoluted XPS spectra in the O 1s region are shown in Fig. 2c. Four peaks were ascribed to oxygen functional groups bound to nanocarbon material. They included: carboxyl groups (COOH) at 534.4 eV, ester groups (O–C=O) at 533.1 eV, hydroxyl groups (C–OH) at 532.1 eV, and carbonyl groups (C=O) at 530.9 eV^31^.

The majority of these signals could be attributed to either hydroxyl, carboxyl, or ester functionalities on the surface of SWCNTs inevitably introduced during the synthesis. It was noted that the relative contribution of ester to hydroxyl groups increased with the use of the samples as HER electrodes. Moreover, peaks attributed to oxygen bound with iron atoms were also found in all the spectra. The signals derived from Fe–O bonds in iron oxides usually located at 530.0 eV were present in each of them. Interestingly, a peak at 531.3 eV was detected exclusively in SWCNT films subjected to HER. This signature can be attributed to Fe-OH bonds in iron hydroxides^32^. Both signals (Fe–O and Fe-OH) increased in intensity as the HER was continued. Therefore, a conclusion was drawn that the enhanced electrochemical performance may come from changes in the chemical state of iron, contaminating the SWCNTs.

The Fe 2p spectra of SWCNT films were collected and studied to investigate the impact of HER on the residual iron present in the material (Fig. 2d). In all the cases, signals were distinguished at 706.9 and 720 eV, which could be attributed to metallic Fe 2p_3/2_ and Fe 2p_1/2_ peaks, respectively. Also, all the samples gave peaks at 711.0 and 724.4 eV, which fitted the energies characteristic for Fe 2p_3/2_ and Fe 2p_1/2_ in Fe_2_O_3_ or other Fe(III) compounds^32,33^. It was clearly seen that the relative intensity of the signals changed with the time of SWCNT film use for HER. The iron oxide signal gradually increased at the expense of metallic iron peaks. The results, therefore, strongly suggested that, during the first 20 h of HER, material activation stemmed from partial oxidation of iron nanoparticles (catalyst residue) into iron oxides and hydroxides.

**Figure 3:**
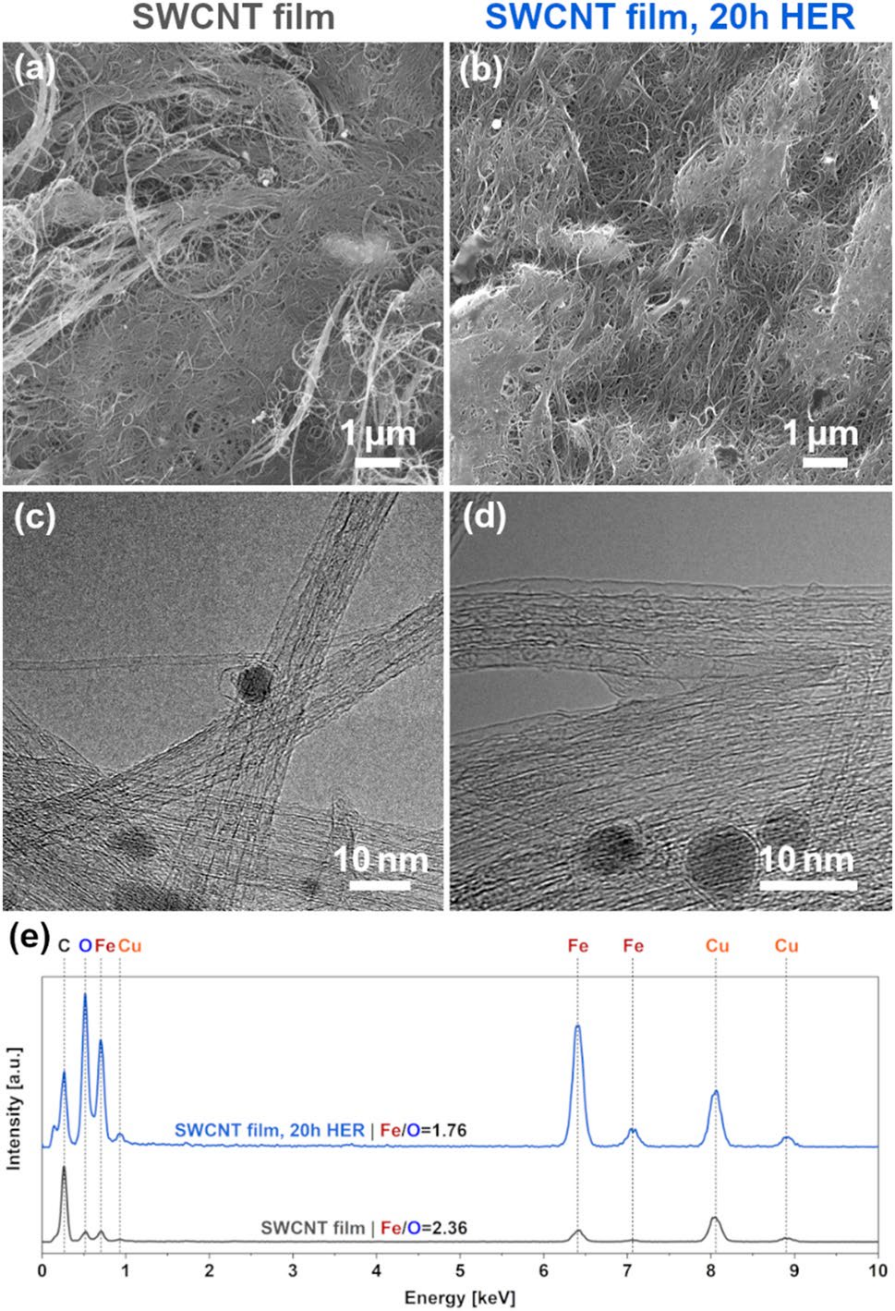
Characterization of SWCNT films (freshly annealed and after 20 h of HER) by (**a**, **b**) SEM, (**c**, **d**) TEM, and (**e**) EDX coupled with TEM (normalized to the maximum of the intensity of carbon peak).

Characterization of the material by SEM and TEM corroborated these findings (Fig. 3a–d). No apparent change to the material's microstructure could be discerned in the SEM micrographs (Fig. 3a, b). Thus, the observed activity increase could not be ascribed to densification of the material, which could improve its electrical conductivity. Still, it appeared to result from modification of metal nanoparticle impurities inevitably present in nanocarbon material. As shown on the TEM micrographs, the films were composed of bundled-up SWCNTs, which did contain the residual iron catalyst coming from the synthesis stage (Fig. 3c, d), which could promote the HER. They can be detected in both the neat SWCNT material and exposed to HER for a prolonged time. Furthermore, the EDX analysis confirmed that the chemical composition of the material was modified during the process (Fig. 3e). The atomic ratio of iron to oxygen changed from 2.36 to 1.76 after HER, which supported the hypothesis that iron was subjected to oxidative activation under these operational conditions.

Three practical notes have to be made. Firstly, Cu was disregarded from consideration as its presence resulted from the application of Cu grids for characterization. Secondly, the relative proportion of C, Cu, and Fe may change from one analysis area to the other. This is because SWCNTs and iron nanoparticles contaminating them are not uniformly deposited on the Cu grids. Furthermore, iron nanoparticles are randomly distributed on the SWCNT bundles. Therefore, once the characterization area is changed, the intensity of these peaks will vary. Nonetheless, the ratio of iron to oxygen should remain similar regardless of the area of investigation in the pure and HER-treated SWCNT films assuming iron is not oxidized. We previously excluded the possibility of SWCNT oxidation, so the observed changes in the Fe/O ratio support the hypothesis of iron oxidation. Thirdly, the detected oxygen could not have originated from the oxygen adsorbed on the surface of TEM grids. We verified it by characterizing the composition of an SWCNT free section of the grid, which showed a negligible amount of oxygen (Fig. S8). In light of these findings, it was concluded that the oxidation of iron nanoparticles gave rise to the observed enhancement of the electrocatalytic activity towards HER of SWCNT film electrodes.

**Figure 4:**
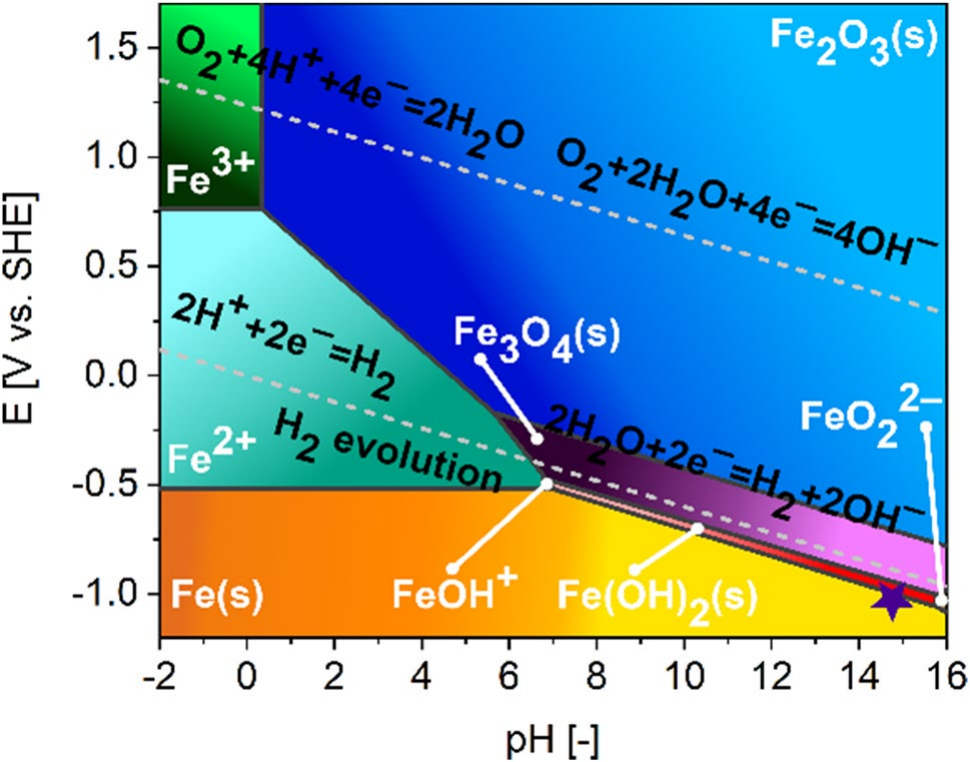
Pourbaix diagram for Fe. Bold lines indicate boundaries between various Fe forms. The area between dashed (water redox) lines highlights conditions under which water is stable. Above and below these lines, water is unstable with respect to O_2_ and H_2_, respectively. HER operational parameters utilized in this work, for which the increase in activity in time was observed, were indicated with a star.

How can iron oxidize at the cathode, where the conditions are sufficiently reducing to obtain hydrogen from water? Fe oxidation resulting in the formation of iron oxide or hydroxide during prolonged electrolysis in 1.0 M KOH can be promoted by the mechanical damage of the microstructure of the film by evolving bubbles^34^, resulting in an increased likelihood of oxidation of Fe(s) to Fe(OH)_2_(s), as the metallic Fe particles are de-encapsulated. Because iron is a rather active metal, especially when nanostructured, the galvanostatic test conditions (bulk solution pH = 14 and potential of ca. − 1.0 V vs. SHE) might not be reducing enough to prevent the corrosion of Fe nanoparticles (see Pourbaix diagram for iron presented in Fig. 4). Taking into account that the pH of unbuffered electrolytes in the vicinity of the cathode during electrolysis can be further elevated by a minimum of 1 unit^35^, the hypothesized transformation is possible from the thermodynamic point of view. Besides that, the ICP-OES characterization results also confirm that Fe's release by the mechanism speculated above is possible as, after 20 h HER treatment, the Fe content in 1.0 M KOH increased from 27.3 to 48.7 ppb. The additional Fe in the liquid must have originated from the SWCNTs. Consequently, we can conclude that despite the slight decrease of Fe amount in the electrode, the electrocatalytic performance increases in time as Fe undergoes oxidation-activation.

The chronopotentiometry electrode test, presented in Fig. S9, further confirmed the validity of the Pourbaix diagram in an acidic medium (0.5 M H_2_SO_4_). In this medium and under such operational conditions, the HER activity of SWCNT film decreased during electrolysis. The deterioration of performance resulted from the leaching of Fe in the form of Fe^2+^. Iron cannot catalyze the HER if it is no longer physically bound to the SWCNT substrate. The result stays in contrast to an earlier report showing an opposite behavior in an acidic medium^36^. The authors attributed this effect to the phenomenon of acid intercalation, which supposedly separated individual SWCNTs in bundles, thereby increasing the surface area available for the HER. SWCNT exposure to acids opened up the microstructure in the referred work, whereas our earlier reports show that porous CNT materials may density when immersed in acidic environments^37^. The SWCNT films used in this study have an appreciable surface area^38^, and condense similarly when put in contact with mineral acids, so the mechanism described by Das et al. is not in force herein. Instead, the gradual loss in HER performance in acidic conditions for our films over time was caused by Fe leaching and densification of the material.

**Figure 5:**
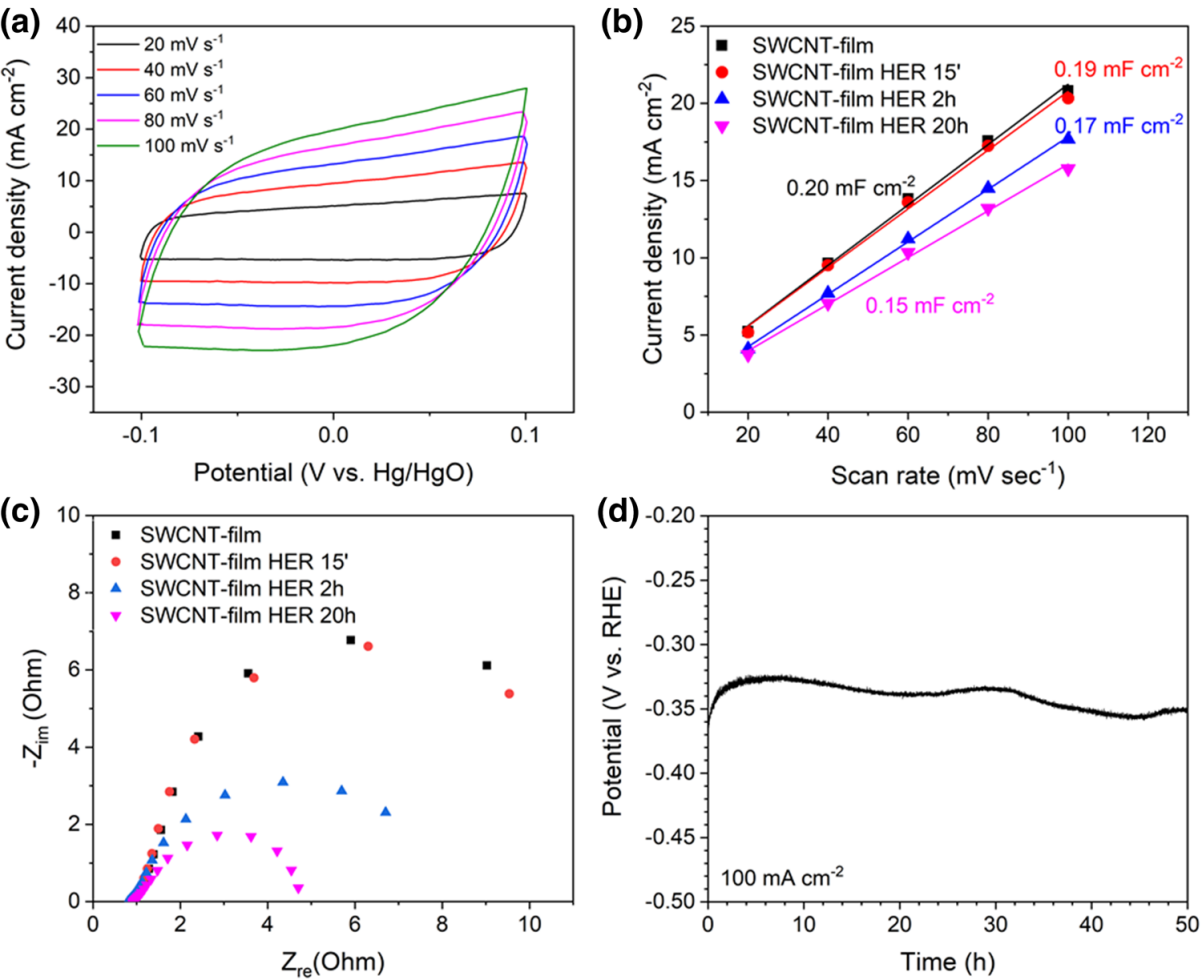
(**a**) Cyclic voltammetry plots at different scan rates for SWCNT film after 20 h of HER, (**b**) linear fits of current densities versus scan rates for freshly annealed SWCNT films and subjected to HER for 15 min, 2 h, and 20 h, (**c**) Nyquist plots for electrodes at different stages of activation at the overpotential of 275 mV, and (**d**) HER chronopotentiometry curve for SWCNT film after 20 h of HER at the current density of 100 mA cm^−2^.

To examine the described electrocatalyst further, the electrodes' electrochemically active surface areas (ECSA) were estimated by means of cycling voltammetry at different stages of activation, assuming that it is directly proportional to the double-layer capacitance of the SWCNT films^39^. In Figure a, the CV plots for SWCNT film after 20 h of HER recorded at different scan rates are presented. The mean values of capacitive currents, extracted from CV curves for all samples, were plotted versus the scan rates. The double-layer capacitances were determined by linear fits of the obtained relationships (Fig. 5b).

The results showed that during the activation process, the electrochemically active surface area of the films gradually decreased from 0.19 to 0.15 mF cm^−2^. Even though ECSA usually correlates positively with electrocatalytic activity in HER^40,41^, the observed small decrease of ECSA did not entail the reduction of the electrocatalytic activity of materials. The loss of ECSA was compensated by a favorable transformation of nanoparticles from iron to iron hydroxide described above.

Furthermore, the EIS spectra were also acquired and presented as Nyquist plots (Fig. 5c). By fitting the proper equivalent circuit^42,43^ to data points (Figure S11), the charge transfer resistance (R_CT_) and uncompensated resistance (R_U_) values were calculated and presented in Table S1. The results showed that the R_CT_ continuously decreased with the SWCNT film activation time from the value of 15.9 to 3.6 Ω, which is manifested by shrinking diameters of recorded semicircles. The reduction of reaction resistance was consistent with the observed overpotential drop during activation. The R_u_ for all samples had a comparable value of ca. 1.1 Ω.

Due to excellent overpotential, SWCNT film after 20 h of HER was also subject to a long-term stability test at a high current density of 100 mA cm^−2^. As shown in Fig. 5d, during the first hours of electrolysis, the overpotential decreased slightly, and later showed slight fluctuations due to differences in temperature during the day and night. Nevertheless, during 50 h of electrolysis under such harsh conditions, the overpotential did not exceed the initial value of c.a. 370 mV, proving excellent durability of the material and suitability for HER.

To sum up, strong HER activity enhancement was observed due to partial oxidation of Fe nanoparticles encapsulated in SWCNTs forming free-standing electrocatalytic films. The distinct activity improvement can be attributed to the optimization of Gibbs free energy of hydrogen adsorption at the surface of the modified nanoparticles, which plays a vital role in HER. Such phenomenon was observed upon slight oxidation of nickel–iron oxide nanoparticles in a recent publication by Suryanto et al.^44^ (experimentally and by DFT calculations). Although, in our case, the content of iron nanoparticles is very low after purification, still the boost to the electrocatalytic activity can be discerned in time while they transform from Fe(s) to Fe(OH)_2_(s) on the surface of SWCNTs. Thus, partial oxidation of iron may give rise to the formation of an analogous Fe/Fe(OH)_2_ interface of improved electrocatalytic activity. Lastly, previous research demonstrated that metal nanoparticles encapsulated in carbon may significantly enrich the charge density of carbon atoms neighboring the nanoparticle, thereby tuning their electronic structure and optimizing the binding energy of HER intermediates^45,46^. The effect intensifies with decreasing number of encapsulating carbon layers^46^, so it should be most pronounced in the case of SWCNTs, which were investigated in this study. This fact may explain the already high electrocatalytic activity of SWCNT films toward HER even before the process itself activates them.

## Conclusions

In summary, we have prepared a high-performance electrocatalytic electrode for application in alkaline HER in the form of the free-standing film composed of SWCNT. During the first 20 h of galvanostatic performance at the current density of − 10 mA cm^−2^, the material undergoes activation, which drops HER overpotential from ca. 300 to 225 mV. Physicochemical characterization of the film at various stages of activation revealed that the activity boost resulted from partial oxidation of residual Fe nanoparticles (used to synthesize SWCNTs), which inevitably contaminate the SWCNTs, to Fe(OH)_2_ species. As a consequence, the more electrocatalytically active Fe(OH)_2_ and Fe/Fe(OH)_2_ clusters were formed. From the reaction mechanism point of view, this modification decreased charge transfer resistance during the electrochemical reaction as the species mentioned above offer optimized binding energy for HER intermediates.

The activated electrode exhibited further excellent stability with negligible activity loss during over a week of galvanostatic performance at the current density of 10 mA cm^−2^ and 50 h of performance at 100 mA cm^−2^. The unique robustness can be attributed to the high-quality constituent SWCNTs, which did not undergo any chemical changes at working conditions, as well as to the free-standing feature of the material, which provided channels for effective transport of the reactants to the electrocatalytically active sites while maintaining high electrical conductivity. These findings pave the way towards engineering high-performance and low-cost electrodes for alkaline HER and provide new insights into electrode properties that determine electrocatalytic performance.

## Supplementary Information

Supplementary Information.
