# Supplementary material for: Revealing the effect of electrocatalytic performance boost during hydrogen evolution reaction on free-standing SWCNT film electrode
Source: Sci Rep. 2021 Oct 7;11:19981. doi: 10.1038/s41598-021-99458-8 (PMC8497545; doi:10.1038/s41598-021-99458-8)
Supplement: Supplementary file 1 — Supplementary Information. [file 41598_2021_99458_MOESM1_ESM.pdf]

Supporting information

Revealing the effect of electrocatalytic performance boost during hydrogen evolution reaction on free-standing SWCNT film electrode

Karolina Kordek-Khalil[a], Dawid Janas[b,*], Piotr Rutkowski[a,*]

[a] Wrocław University of Science and Technology, Department of Process Engineering and Technology of Polymer and Carbon Materials, Wybrzeże Wyspiańskiego 27, 50-370, Wrocław, Poland

[b] Department of Organic Chemistry, Bioorganic Chemistry and Biotechnology, Silesian University of Technology, B. Krzywoustego 4, 44-100 Gliwice, Poland

*Corresponding author(s): Dawid.Janas@polsl.pl, Piotr.Rutkowski@pwr.edu.pl

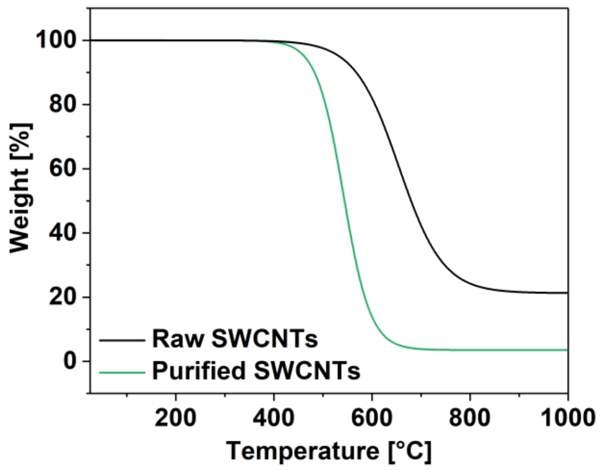

Figure S1 Thermograms of raw and purified SWCNTs obtained by TGA.

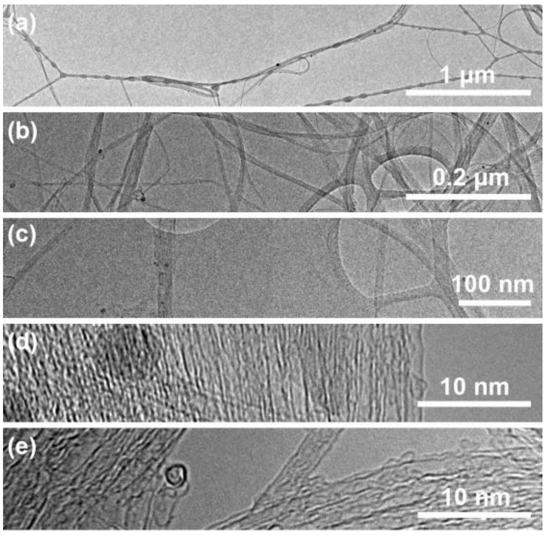

Figure S2 TEM micrographs of purified SWCNTs showing that a small amount of Fe is still present after the processing. Panel (e) visualizes a front of a CNT, confirming that the material is single-walled.

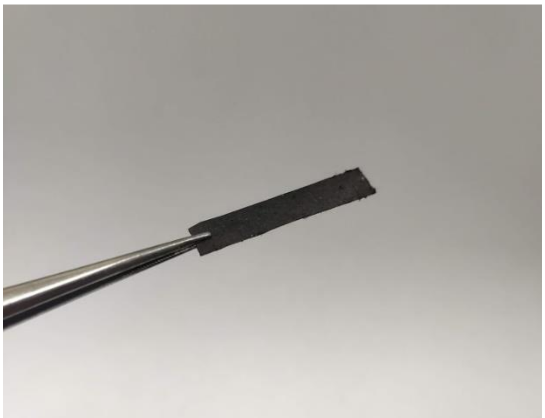

Figure S3 A photograph of a strip of a free-standing SWCNT film used to prepare the electrodes for HER (5 mm x 25 mm).

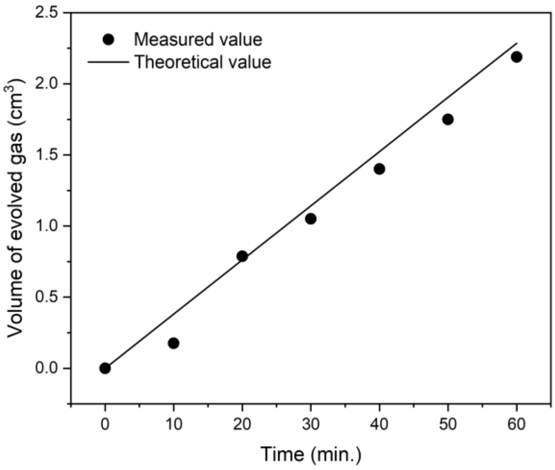

Figure S4 Experimental and theoretical volumes of H_2 evolved from SWCNT-film 20h electrode at a current density of 10 mA cm^{-2}, measured for 60 min.

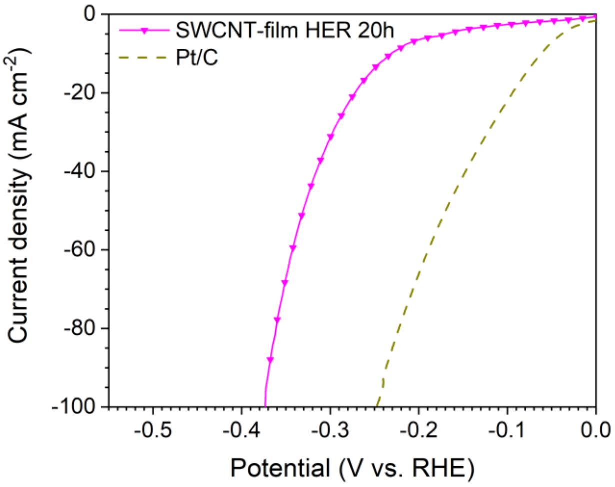

Figure S5 LSV plot for SWCNT-film 20h electrode and Pt/C benchmark electrocatalyst.

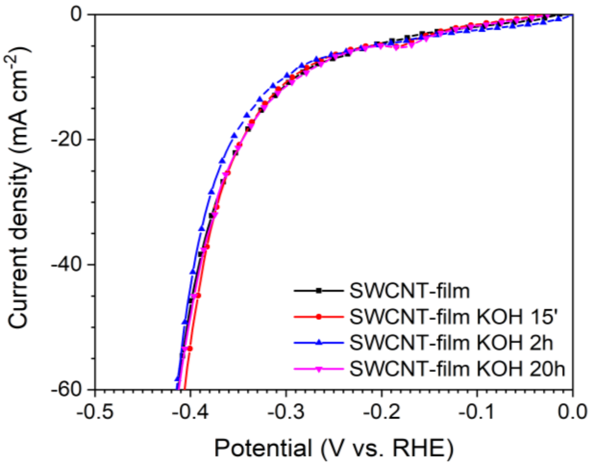

Figure S6 LSV plots for HER for fresh SWCNT film and SWCNT film samples after immersing the material in 1.0 M KOH for 15 min., 2h, and 20h.

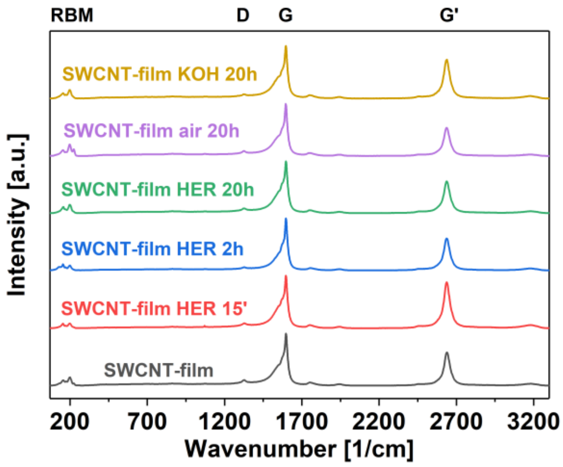

Figure S7 Raman spectra of SWCNT films after HER for the specified time or after the exposure to air/KOH for 20h.

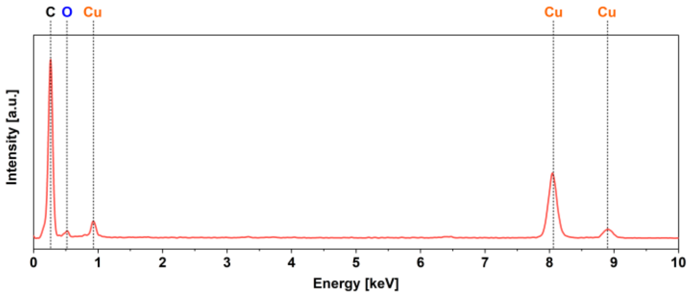

Figure S8 EDX spectrum of an SWCNT free section of the TEM grid (holey carbon film/Cu).

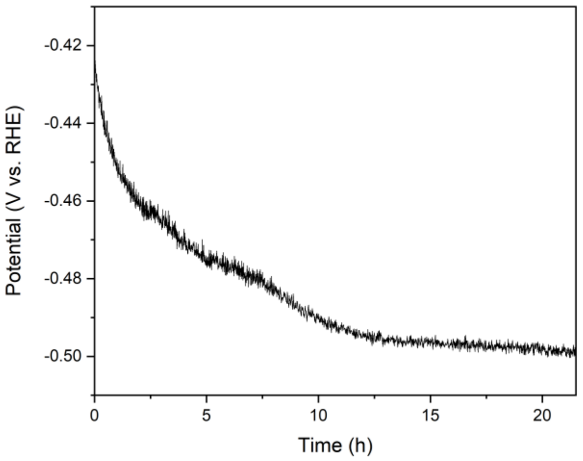

Figure S9 Chronopotentiometry plot at the current density of -10 mA cm^{-2} for SWCNT film in an acidic electrolyte (0.5 M H_2SO_4).

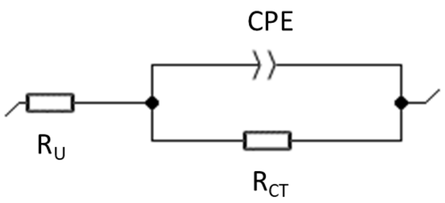

Figure S10 Equivalent circuit applied for fitting the EIS data.

Table S1 Summarized resistance values derived from Nyquist plots.

Electrode	R_u [Ω]	R_{ct} [Ω]
SWCNT-film	1.1	15.9
SWCNT-film HER 15'	1.0	13.5
SWCNT-film HER 2h	1.0	7.0
SWCNT-film HER 20h	1.2	3.6
